# An image encryption scheme based on public key cryptosystem and quantum logistic map

**DOI:** 10.1038/s41598-020-78127-2

**Published:** 2020-12-03

**Authors:** Guodong Ye, Kaixin Jiao, Xiaoling Huang, Bok-Min Goi, Wun-She Yap

**Affiliations:** 1 Faculty of Mathematics and Computer Science, Guangdong Ocean University, Zhanjiang, 524088 China; 2 Lee Kong Chian Faculty of Engineering and Science, Universiti Tunku Abdul Rahman, Jalan Sungai Long, 43000 Cheras, Selangor Malaysia

**Keywords:** Computer science, Information technology

## Abstract

Most of existing image encryption schemes are proposed in the spatial domain which easily destroys the correlation between pixels. This paper proposes an image encryption scheme by employing discrete cosine transform (DCT), quantum logistic map and substitution-permutation network (SPN). The DCT is used to transform the images in the frequency domain. Meanwhile, the SPN is used to provide the security properties of confusion and diffusion. The SPN provides fast encryption as compared to the asymmetric based image encryption since operations with low computational complexity are used (e.g., exclusive-or and permutation). Different statistical experiments and security analysis are performed against six grayscale and color images to justify the effectiveness and security of the proposed image encryption scheme.

## Introduction

With the advancement of social media and the development of Internet, transmission of images becomes a new normal given that images are more visual and informative. Thus, it raises the need for image encryption techniques to protect the confidentiality of images. When proposing an image encryption technique, it is necessary to consider the unique attributes of digital images, such as high redundancy and strong correlation between adjacent pixels. Due to the unique attributes, traditional cryptographic techniques are no longer suitable for image encryption^1^.

Different techniques were introduced to resolve the problems arisen from the unique image characteristics. The techniques are based on chaos theory^2–4^, compressed sensing^5–7^, DNA coding^8–10^ and quantum communication^11–13^. Out of these techniques, chaos theory introduced by Lorenz^14^ is widely used for image encryption due to the states of chaotic systems are apparently random and irregular under the control of highly sensitive initial values. The chaotic maps widely used for image encryption are logistic map, logic-sine-coupling map, sine map and Chebyshev map.

Along the direction of chaos theory, Sui et al.^15^ proposed a double-image encryption method based on optical interference and logistic map, which effectively avoids the contour problem in the interference encryption method. Hua et al.^16^ proposed a two-dimensional logic-sine-coupling map and applied the proposed map to a classical confusion-diffusion framework for image encryption. Other than two-dimensional chaotic map, Pak and Huang^17^ proposed a new color image encryption algorithm by utilizing three one-dimensional chaotic maps including logistic, sine and Chebyshev. Even though low-dimensional chaotic map is simple and easy to implement, it suffers from the drawback of limited range of chaotic behavior and smaller key space. Thus, multiple low-dimensional or high-dimensional chaotic maps are exploited to form an image encryption scheme with higher security by enlarging the key space^18–22^. For example, Chen et al.^19^ proposed an optical image encryption scheme combining image scrambling and random coding based on the three-dimensional chaotic system to solve the shortcomings of typical image scrambling codes. Besides, Chai et al.^23^ proposed an efficient image compression and encryption scheme by combining hyperchaos system with two-dimensional compressive sensing, and realized the functions of compression and encryption at the same time, which overcame the risk of using nonlinear transformation in low-dimensional chaos system. Recently, low-dimensional chaotic system is used to construct a parallelizable image encryption scheme as the computational cost is lower^24,25^.

Due to the breakthrough of quantum teleportation and quantum computing, quantum image processing has also received increasing attention in the field of information security. The image types will be first mapped to quantum domain before performing quantum computation. Zhou et al.^26^ designed a quantum version of the generalized Arnold transform. Subsequently, a quantum image encryption algorithm was proposed based on the generalized Arnold transform and double random phase encoding technique. Meanwhile, Luo et al.^27^ proposed an image encryption scheme based on quantum coding. The classical digital image data is mapped to the quantum state, which ensures the accurate extraction of the classical image. At the same time, quantum adjacency switching reduces computational complexity.

The aforementioned image encryption algorithms are constructed in the spatial domain which easily destroys the correlation between pixels^28^. The frequency domain based image encryption scheme is more efficient, more robust and preserve the information of image even though going through the inverse process. Frequency domain based image encryption normally involves fractional Fourier transform, discrete wavelet transform, discrete cosine transform and Gabor transform. Kong and Shen^29^ proposed a multiple-image encryption scheme based on multichannel fractional Fourier transform and wavelet transform. The increase of the number of encrypted images solves the problem of insufficient capacity and improves the flexibility and variability of the scheme. To reduce the amount of encrypted images and tolerate a certain range of noise intensity, Zhou et al.^30^ proposed an efficient dual image encryption scheme based on discrete wavelet transform and discrete fractional random transformation. On the other hand, Chen et al.^31^ proposed an image encryption method in fractional domain through singular value decomposition and Arnold conversion.

Different from other symmetric key based image encryption schemes, Dong^32^ designed an asymmetric color image encryption scheme based on the discrete mapping. The hash value and the initial number of iterations are used as keys in the encryption process. In the decryption process, the three initial values of piecewise linear chaotic map and the initial number of iterations are used as keys. Along the same direction, Wu et al.^5^ combined compressed sensing and cylindrical diffraction techniques to propose an asymmetric multiple-image encryption algorithm to prevent information leakage and phase retrieval attacks. Besides, the use of compressed sensing aims to compress the amount of data in multiple-image encryption schemes. Lastly, Liu et al.^33^ proposed an asymmetric color image encryption scheme based on a four-wing complex chaotic system. The 512-bit hash of the image is used to generate one-time initial conditions, and then the red, green and blue components of the odd and even indexes are encrypted respectively. It can be observed that the aforementioned asymmetric image encryption schemes are all based on the frequency domain.

This paper aims to propose a secure yet sensitive image encryption scheme for the secure transmission of images. To achieve this aim, we first apply public key cryptosystem to generate the initial values of the quantum logistic map. The initial value generation method can be extended to other image encryption schemes too. For illustration purposes, RSA cryptosystem^34^ is selected as the underlying public key cryptosystem. With the knowledge of initial values, pseudo-random keystream sequences can be generated using the chaotic map. A three-dimensional quantum logistic map is utilized to ensure a sufficient large key space to prevent the brute force attack. The keystream generated will then be utilized to provide the properties of confusion and diffusion by using a substitution–permutation network that consists of row permutation, column permutation and substitution. Besides, the discrete cosine transform is used to transform the images into the frequency domain. To improve the security of the proposed image encryption scheme, the quantum logistic map is employed due to its larger key space and better chaotic behaviour. Besides, SPN needs to be iterated for 5 rounds in total to resist against differential-like attacks. Different statistical experiments and security analysis are performed on different grayscale and color images to justify the effectiveness and the security of the proposed image encryption scheme.

The rest of this paper is organized as follows. Section 2 briefly introduces some basic knowledge of public key cryptosystem, discrete cosine transform and quantum logistic chaotic map. Section 3 presents the proposed image encryption scheme. Section 4 presents the experimental results and its analysis against different attacks or security concerns. Finally, Section 5 gives the concluding remarks.

## Preliminaries

### Public key cryptosystem

Public key cryptosystem is an asymmetric encryption consisting of the following three algorithm:

- **Key generation**, Gen($1^k$): Given a security parameter *k*, outputs a pair of keys, namely public key *pk* and secret key *sk* respectively.
- **Encryption**, Enc(*pk*, *m*): Given the input *pk* and message *m*, outputs the ciphertext *c*.
- **Decryption**, Dec(*sk*, *c*): Given the input *sk* and ciphertext *c*, outputs the message *m*.

A public key cryptosystem is said to fulfill correctness if $\forall m,pk,sk$, Dec(*sk*,  Enc(*pk*, *m*)) $= m$ where (*pk*, *sk*) $\leftarrow$ Gen($1^k$). For illustration purposes and full description, RSA cryptosystem^34^ is selected as the underlying public key cryptosystem.

### Quantum logistic map

Goggin et al.^35^ derived a logistic map with quantum corrections by coupling a kicked quantum system to a bath of harmonic oscillators. The proposed quantum logistic map was applied to image encryption by Akhshani et al.^36^ as follows:

1 $$\begin{aligned} x_{n+1}&= r(x_n-|x_n|^2)-ry_n, \end{aligned}$$

2 $$\begin{aligned} y_{n+1}&=-y_ne^{-2\beta }+e^{-\beta }r[(2-x_n-x_n^*)y_n-x_nz_n^*-x_n^*z_n], \end{aligned}$$

3 $$\begin{aligned} z_{n+1}&=-z_ne^{-2\beta }+e^{-\beta }r[2(1-x_n^*)z_n-2x_ny_n-x_n], \end{aligned}$$

where $x=\langle a \rangle ,y=\langle \delta a^\dagger \delta a\rangle ,z=\langle \delta a \delta a\rangle$ and $\beta$ is dissipation parameter. Besides, $x_n,y_n$ and $z_n$ are complex numbers with $x_n^*$ being the complex conjugate of $x_n$. The same notation applies to $x_n$. However, the initial values $x_n$, $y_n$ and $z_n$ are set as real numbers to meet the requirement of communication.

**Figure 1:**
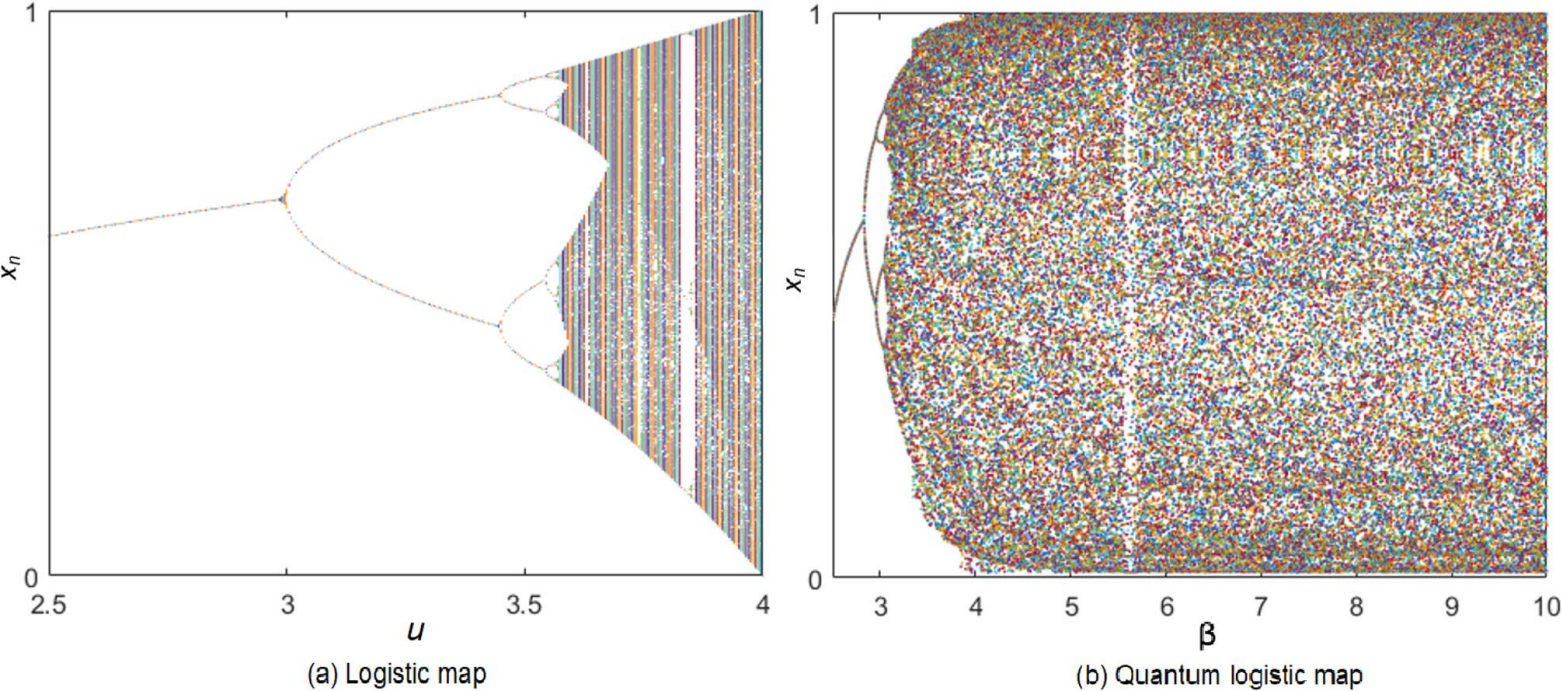
Bifurcation.

Meanwhile, the logistic map is written as follows:

4 $$\begin{aligned} x_{n+1}= ux_n(1-x_n), \end{aligned}$$

where $x_n\in [0,1]$ and $u\in [0,4]$. We analyse both logistic map and quantum logistic map in terms of bifurcation, Lyapunov exponent and phase diagram. Bifurcation is a qualitative change in the dynamics of a given chaotic system due to the change of the control parameter. In the bifurcation diagram, the dotted line shows the chaotic behavior of the system, while the solid line shows that the system has changed to be periodic. Figure 1a shows that the logistic map has chaotic behavior when *u* is in the interval of 3.57–4. In Fig. 1b, when we fix $r=3.99$, the quantum logistic map has a wider chaotic region as the data outputs of the chaotic sequence fully occupies the interval of 0 to 1, when the control parameter $\beta > 4$. As compared with the logistic map, the quantum logistic map has many good properties: (1) Larger key space with a three-dimensional system; (2) Larger continuous interval; (3) More uniform distribution for the output. Therefore, the non-periodicity and randomness of chaotic sequences are enhanced. We like to note that Fig. 1 is drawn by author Wun-She Yap.

Lyapunov Exponent (LE) is a tool to measure the sensitivity of the chaotic map to the slight changes in the initial conditions and control parameters. The LE of a differentiable non-linear system $x_{i+1}=f(x_i)$ can be calculated as:

5 $$\begin{aligned} \lambda =&\lim _{n\rightarrow \infty } \dfrac{1}{n} \sum _{i=0}^{n-1} \text {ln} |f'(x_i)|. \end{aligned}$$

**Figure 2:**
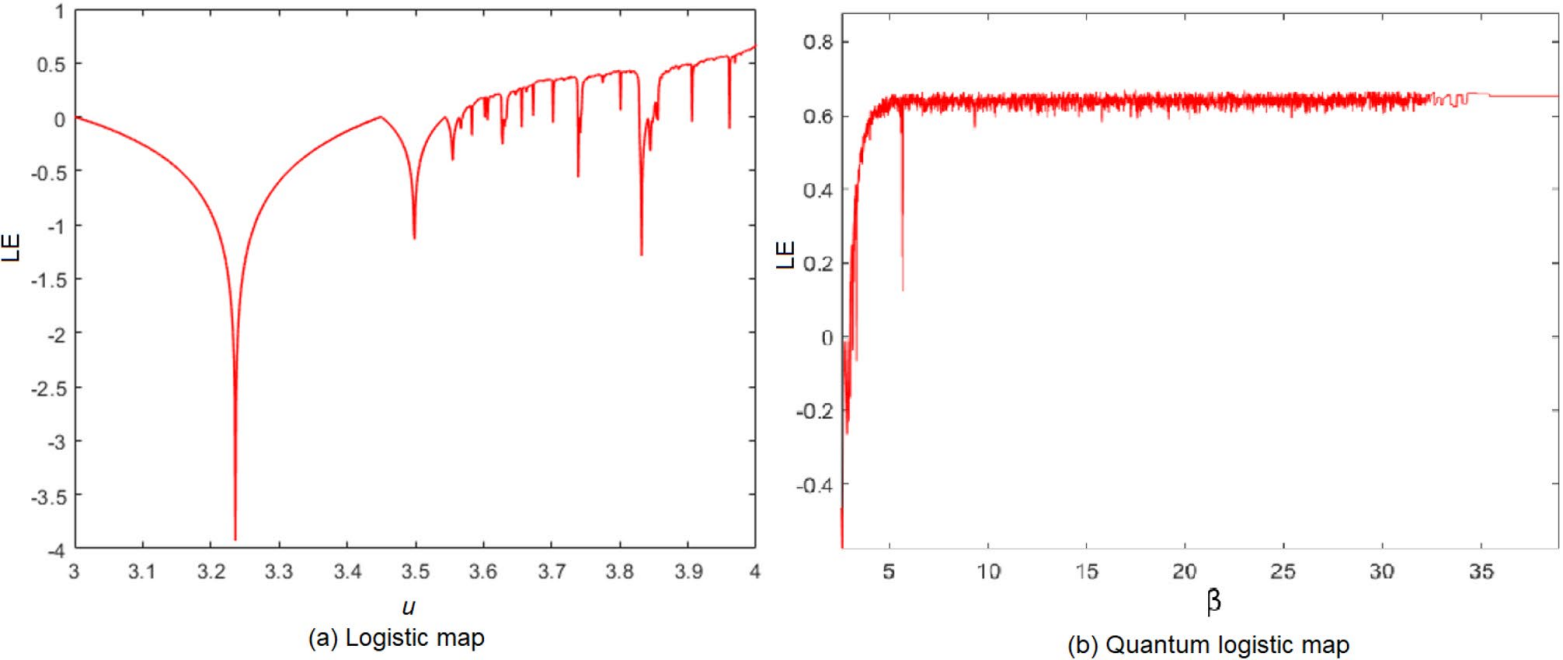
Lyapunov exponent.

The chaotic map with a positive LE demonstrates good chaotic behavior. The higher the LE value indicates a better sensitivity of the map to its initial value. Figure 2 shows that logistic map only have positive LE when $u\in [3.57,4]$ while quantum logistic map has positive LE when $\beta >6$. Therefore, it shows that quantum logistic map has better chaotic behavior than the logistic map. We like to note that Fig. 2 is drawn by author Wun-She Yap.

**Figure 3:**
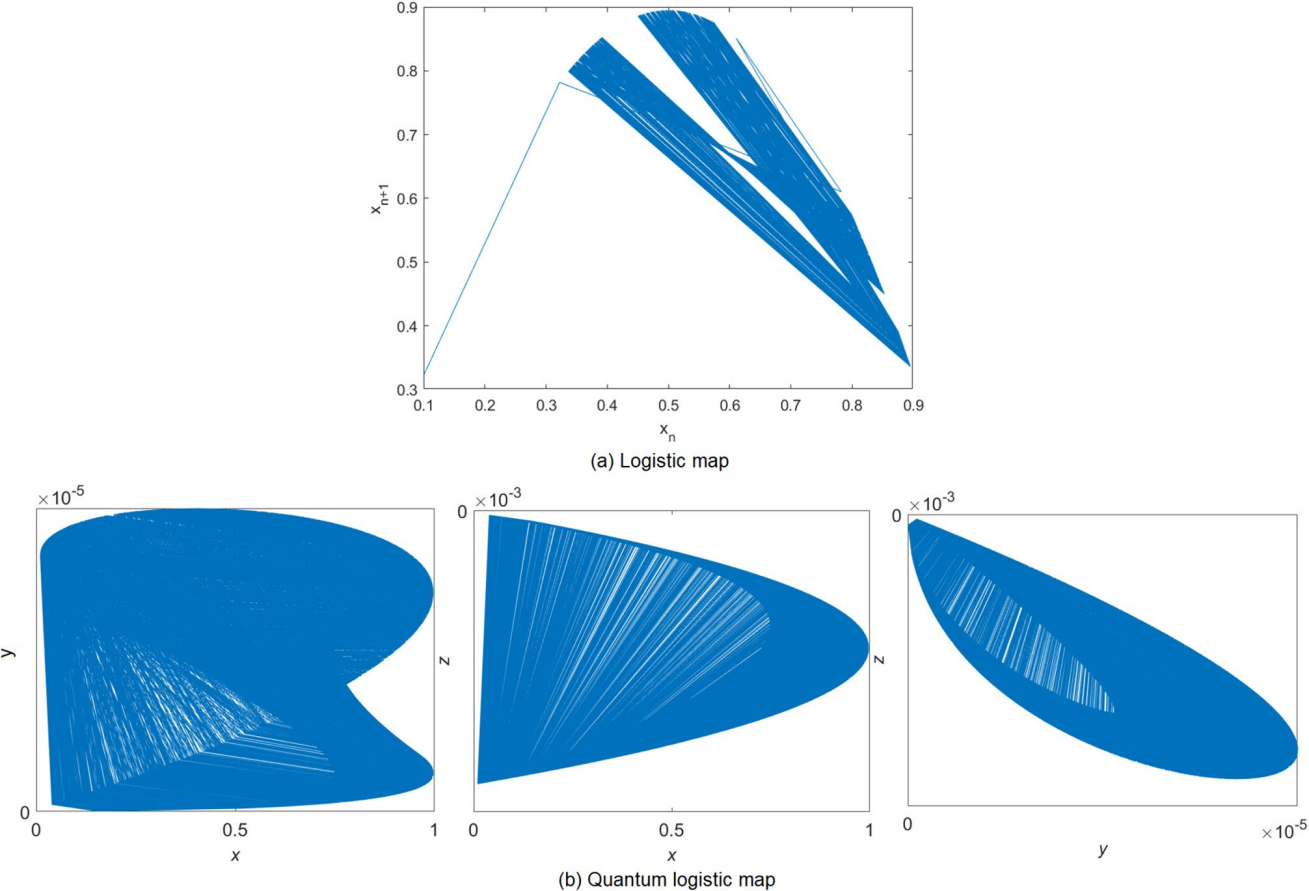
Phase diagram.

For a dynamical system, the distribution of the trajectory on the phase plane demonstrates the randomness of the outputs. Figure 3b shows the phase diagrams of quantum logistic map with the parameters $r=3.99$ and $\beta =7$. As compared to the phase diagram of the logistic map with $u=3.58$ shown in Fig. 3a, the trajectory of quantum logistic map disperses widely on the $x-y$ plane with high density, which indicates that it has a good ergodicity property. We like to note that Fig. 3 is drawn by author Wun-She Yap.

### Discrete cosine transform

Discrete cosine transform (DCT) is widely used for image and video compression standards including JPEG and MPEG. In this paper, the two-dimensional forward DCT^37^ is used. Given an image *P* consisting *M* rows and *N* columns and *P*(*i*, *j*) denotes the pixel value of image *P* at row *i* and column *j*, the DCT of image *P* is defined as follows:

6 $$\begin{aligned} F(u,v) = \sigma (u)\sigma (v)\sum _{i=0}^{M-1}\sum _{j=0}^{N-1}P(i,j)\text {cos}\left[ \dfrac{(2i+1)\pi }{2M}u\right] \text {cos}\left[ \dfrac{(2j+1)\pi }{2N}v\right] , \end{aligned}$$

where $\sigma (u)$ and $\sigma (v)$ are defined as follows:

7 $$\begin{aligned} \sigma (u) = \left\{ \begin{array}{c} \sqrt{\dfrac{1}{M}}, u = 0\\ \sqrt{\dfrac{2}{M}}, u \ne 0 \end{array} \right. \sigma (v) = \left\{ \begin{array}{c} \sqrt{\dfrac{1}{N}}, v = 0\\ \sqrt{\dfrac{2}{N}}, v \ne 0 \end{array} \right. . \end{aligned}$$

After the DCT transformation, the low-frequency coefficient reflects the outline and gray distribution characteristics of the target in the image while the high-frequency coefficient reflects the detailed information of the target shape. Finally, the image *P* can be restored by the inverse DCT as follows:

8 $$\begin{aligned} P(i,j) = \sigma (u)\sigma (v)F(u,v)\text {cos}\left[ \dfrac{(2i+1)\pi }{2M}u\right] \text {cos}\left[ \dfrac{(2j+1)\pi }{2N}v\right] . \end{aligned}$$

## The proposed image encryption scheme

The proposed image encryption generates the initial values $x_0,y_0$ and $z_0$ based on a plain image *P*, a secret (denoted as message *m*) and ciphertext *c* generated by a secure public key encryption scheme. The proposed initial value generation method removes the need for a secure channel for the transmission of secrets. Besides, the initial value generation method is dynamic since it relies on the ciphertext and image to be encrypted. The proposed image encryption scheme mimics one-time pad^38^ if different secret *m* is selected in each image encryption process. The encryption structures is a substitution-permutation network that iterates for five rounds. Each round consists of permutation (i.e. row and column permutations) and substitution (i.e. exclusive-or operation) to provide the properties of confusion and diffusion.

**Figure 4:**
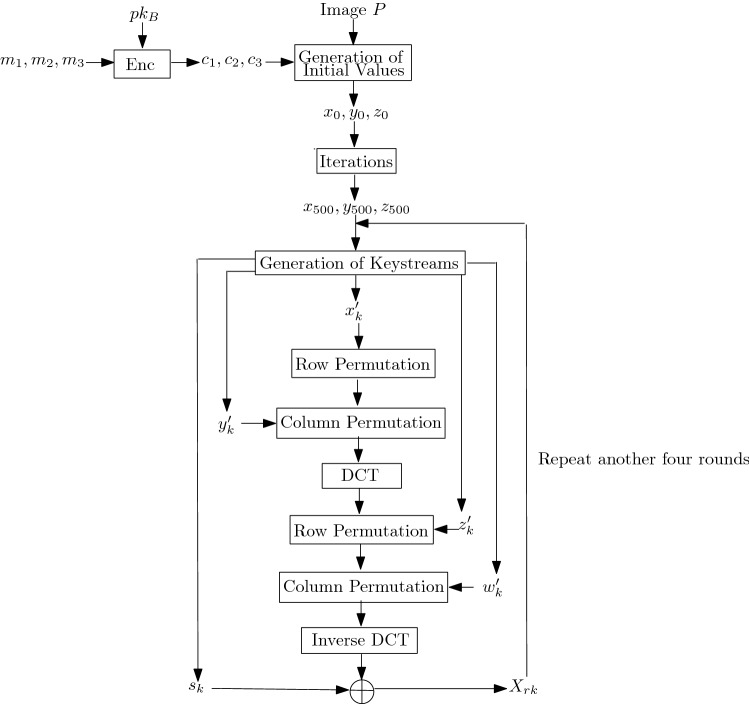
The overview of the proposed image encryption scheme.

Assume the recipient *B* with his public and secret keys $(pk_B,sk_B)$ respectively. *A* wishes to encrypt an image *P* containing $M \times N$ pixels. As shown in Fig. 4, the image encryption process consists of the following steps:

1. Generation of initial values $(x_0,y_0,z_0)$:
  - *A* computes $$r = \sum _{i=1}^M\sum _{j=1}^N \root 5 \of {(P(i,j)+i+j)^2}$$.
  - For $k=1,2,3$, *A* randomly selects three secret messages $m_k$ and generates ciphertexts $c_k=Enc(pk_B,m_k)$. Notice that $m_k\in \{0,1\}^n$ and *n* depends on the public key encryption used.
  - *A* generates the initial values for quantum logistic maps as follows:

- Compute $x_0 = \dfrac{1}{|m_1-c_1|+r}$ where $|\cdot |$ is the modulus function.
- Compute $$y_0 = \dfrac{1}{|m_2-c_2|+r}$$.
- Compute $$z_0 = \dfrac{1}{|m_3-c_3|+r}$$.

(d) *A* computes $x_{500}, y_{500}$ and $z_{500}$ by iterating Eqs. (1), (2) and (3) 500 times with the initial values of $(x_0,y_0,z_0)$. Notice that The first 500 iterations are discarded for each sequence.

2. Encryption round: Let $X_0 = P$. For $rk=0,1,2,3,4$, do:
  - *A* computes $x_{500+rk(MN)+1}, x_{500+rk(MN)+2}, \ldots , x_{500+rk(MN)+MN}$ by applying $x_{500+rk(MN)}$ to Eq. (1).
  - *A* computes $y_{500+rk(MN)+1}, y_{500+rk(MN)+2}, \ldots , y_{500+rk(MN)+MN}$ by applying $y_{500+rk(MN)}$ to Eq. (2).
  - *A* computes $z_{500+rk(MN)+1}, z_{500+rk(MN)+2}, \ldots , z_{500+rk(MN)+MN}$ by applying $z_{500+rk(MN)}$ to Eq. (3).
  - *A* computes $$x'_k = \text {floor}(x_{k+500+rk(MN)} \times 10^{14})\text {mod} \, N +1$$ for $$k=1,2,\ldots , M$$.
  - *A* computes $$y'_k = \text {floor}(y_{k+500+rk(MN)} \times 10^{14})\text {mod} \, M +1$$ for $$k=1,2,\ldots , N$$.
  - *A* compute $$z'_k = \text {floor}((z_{k+500+rk(MN)}\times 0.6 + x_{k+500+rk(MN)}\times 0.4) \times 10^{14}) \text {mod} \, N +1$$ for $$k=1,2,\ldots , M$$.
  - *A* compute $$w'_k = \text {floor}((z_{k+500+rk(MN)}\times 0.6 + y_{k+500+rk(MN)}\times 0.4) \times 10^{14}) \text {mod} \, M +1$$ for $$k=1,2,\ldots , N$$.
  - *A* computes $$s_{k} = \text {fix}(x_{500+k+rk(MN)}+y_{500+k+rk(MN)}+z_{500+k+rk(MN)})\times 10^{14} \text {mod}\, 256$$ for $$k=1,2,\ldots , M\times N$$.
  - *A* performs the row permutations (i.e. rotate right by $x'_i$ positions for each row *i*) by computing $X'_{rk}(i,j) \leftarrow X_{rk}(i, (j+x'_i-1)\, \text {mod} \, N+1)$ for $i=1,2,\ldots M$ and $j=1,2,\ldots N$.
  - *A* performs the column permutations (i.e. rotate down by $y'_i$ positions for each column *j*) by computing $X''_{rk}(i,j) \leftarrow X'_{rk}((i+y'_i-1)\, \text {mod} \, M+1, j)$ for $i=1,2,\ldots M$ and $j=1,2,\ldots N$.
  - *A* computes the discrete cosine transform coefficient matrix *F* by applying Eq. (6) on $X''_{rk}(i,j)$.
  - *A* performs the row permutations (i.e. rotate right by $z'_i$ positions for each row *i*) by computing $F'(i,j) \leftarrow F(i, (j+z'_i-1)\, \text {mod} \, N+1)$ for $i=1,2,\ldots M$ and $j=1,2,\ldots N$.
  - *A* performs the column permutations (i.e. rotate down by $w'_i$ positions for each column *j*) by computing $F''(i,j) \leftarrow F'((i+w'_i-1)\, \text {mod} \, M+1, j)$ for $i=1,2,\ldots M$ and $j=1,2,\ldots N$.
  - *A* computes the inverse of discrete cosine transform coefficient matrix *G* by applying Eq. (8) on $F''(i,j)$.
  - *A* generates the encrypted image for round *rk* by $(X_{rk+1})_i = G_i \oplus s_i \oplus (X_{rk+1})_{(i-1)}$, $i=1,2,\ldots , MN$, where, $(X_{rk+1})_i$, $G_i$, and $s_i$ denote the *i* elements in $X_{rk+1}$, *G*, and *s* respectively.
3. *A* transmits $c_1,c_2,c_3, r$ and the encrypted image $X_{5}$ to *B*.

**Remark**. We ignore the description of the image decryption process as the decryption process is the inverse of the encryption process and straightforward.

## Experimental results

**Figure 5:**
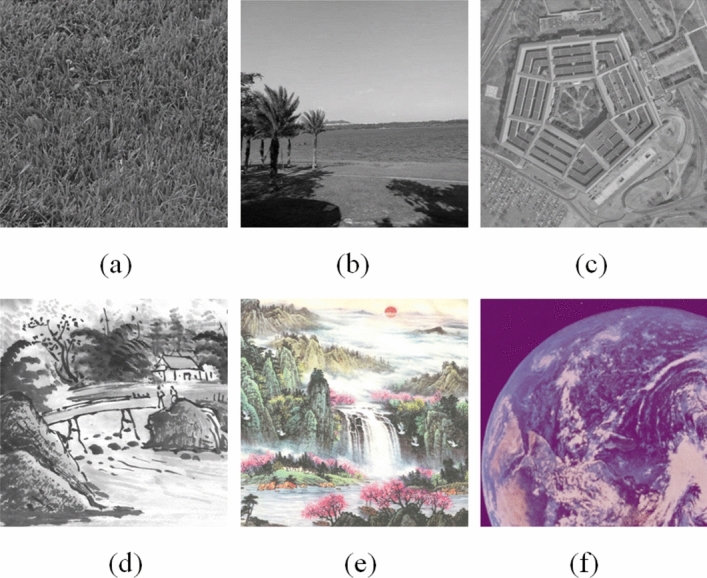
Plain images: (**a**) Grass; (**b**) Tree; (**c**) Pentagon; (**d**) Art; (**e**) Sun; (**f**) Earth.

For illustration purposes, RSA^34^ is selected as the public key encryption scheme. Besides, as shown in Fig. 5, six images are randomly selected (Images Grass, Pentagon and Earth are free from the USC-SIPI database, anyone can read the Copyright Information for these images at http://sipi.usc.edu/database/copyright.php. Image Tree is taken by author Kaixin Jiao. Images Art and Sun are taken by author Guodong Ye) for testing purposes. All the experiments are performed on MATLAB R2017b where the proposed algorithm consists of 5-round encryption process.

### Correctness analysis

Table 1 shows the selected parameters for RSA cryptosystem. Details of RSA can be referred to Ref.^34^. Besides, the selected messages are $m_1 = 5, m_2 = 11$ and $m_3=20$ for illustration purposes. $\beta$ and *r* of quantum logistic map is set as 6 and 3.99, respectively.

**Table 1:** Selected parameters for RSA cryptosystem.

Parameter	*n*	*p*	*q*	*e*	*d*
Value	3317659	1777	1867	121	438217

**Figure 6:**
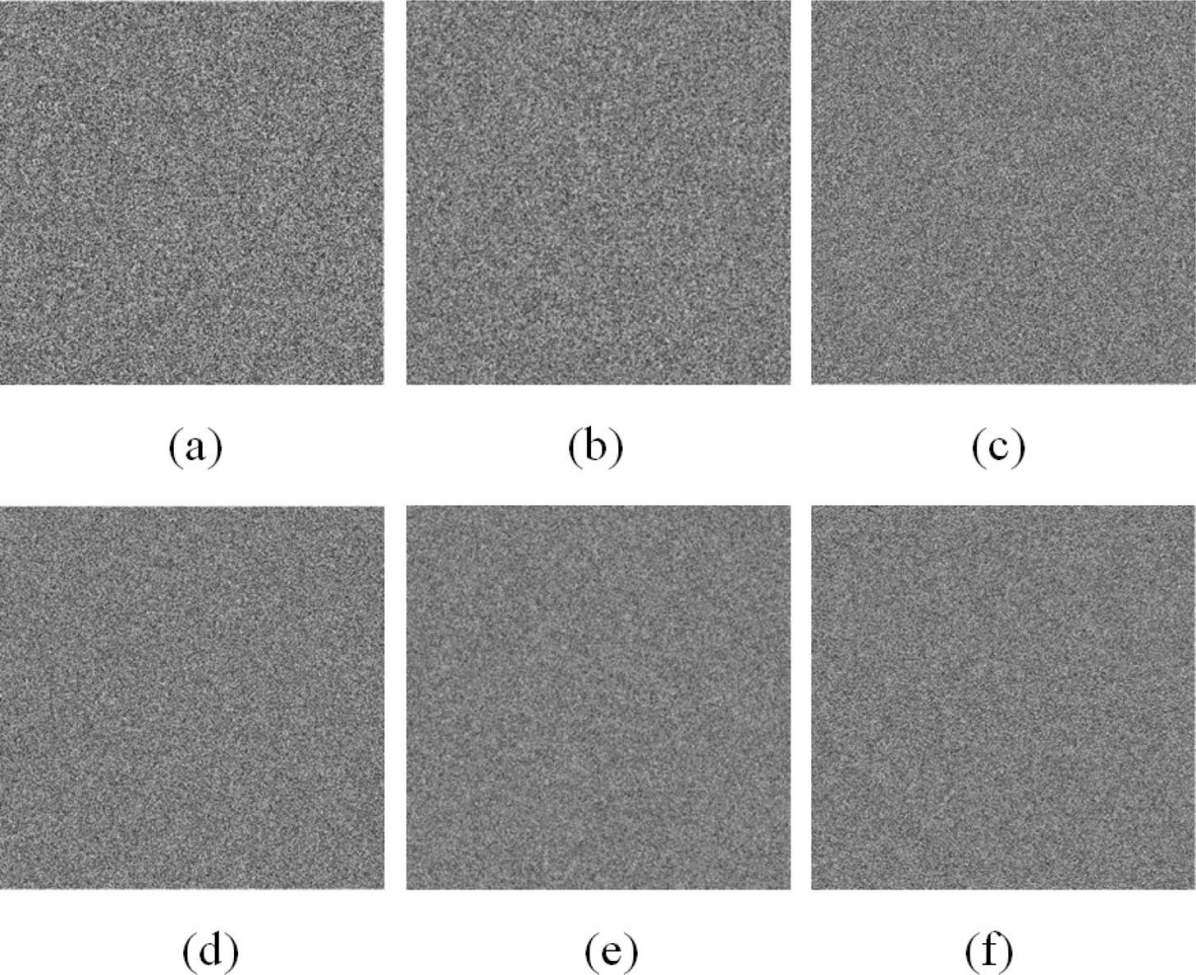
Encrypted images: (**a**) Grass; (**b**) Tree; (**c**) Pentagon; (**d**) Art; (**e**) Sun; (**f**) Earth.

**Figure 7:**
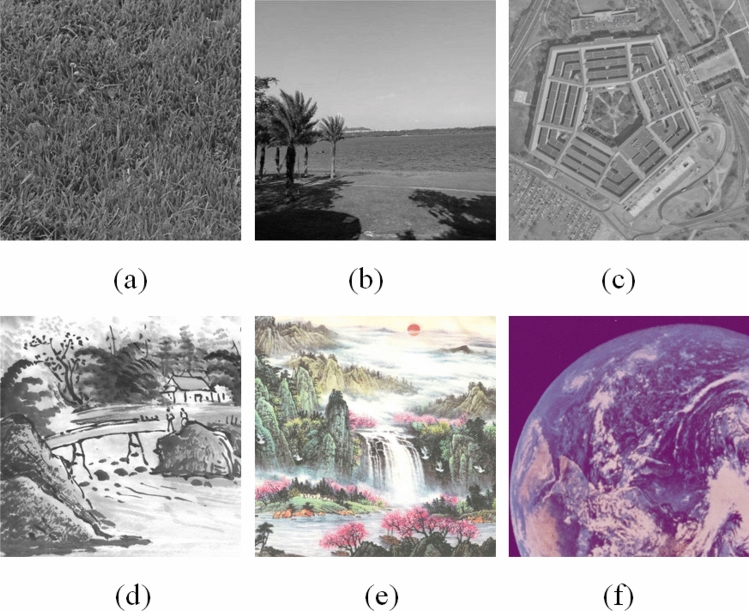
Decrypted images: (**a**) Grass; (**b**) Tree; (**c**) Pentagon; (**d**) Art; (**e**) Sun; (**f**) Earth.

Figure 6 shows the encrypted images of plain images shown in Fig. 5. Notice that as shown in Fig. 7 the image encryption scheme fulfills the correctness properties where the decrypted images are similar to the plain image.

On the other hand, Table 2 presents the time needed by the proposed scheme to encrypt images with different sizes for the different number of rounds. The time measured includes the process to generate the initial value for the underlying quantum logistic map.

**Table 2:** The encryption time needed by the proposed scheme.

Image size	Encryption time (s)				
	1 round	2 rounds	3 rounds	4 rounds	5 rounds
$256 \times 256$	0.018051	0.036021	0.054024	0.064756	0.072107
$512 \times 512$	0.032843	0.065897	0.117721	0.147734	0.181816
$1024 \times 1024$	0.071392	0.132049	0.181668	0.256847	0.441728

### Security and key space analysis

A naive approach to break the proposed image encryption scheme is to guess the initial values $(x_0,y_0,z_0)$. As each initial value is of 14 decimal places with the range between 0 to 1, there exists $10^{14}$ possible values for each of $(x_0,y_0,z_0)$. This contributes to $2^{46.5}$ possible guesses of value $x_0$. This applies to $y_0$ and $z_0$ as well. Thus, there are $2^{139.5}$ possible values of $(x_0,y_0,z_0)$. This also indicates the key space is of 139.5-bit. Besides, the inclusion of *c* in generating initial values is to provide more possibilities by using subtraction and modulus operations. Notice that *r* is with the smaller range given that there exists a smaller number of pixels and each pixel is of 8-bit long^39^. Thus, the generation of initial values will not be greatly affected by *r*.

The second approach is to break the proposed image encryption scheme by breaking the underlying public key encryption scheme. Assuming a public key encryption is secure and with the 128-bit security level, then the second approach will not work.

Instead of recovering the initial values $(x_0,y_0,z_0)$, the adversary may recover the keystreams (also known as round keys). As shown in Fig. 4, the round keys affect the permutations (i.e. row and column) and substitution (i.e. exclusive-or operation). Instead of guessing $x'_k,y'_k,z'_k$ and $w'_k$, it is sufficient for the adversary to guess the permutation directly especially when the image contains a smaller number of pixels. Assuming the image is of $M\times N$ pixels, there exists possible $(M\times N)!$ permutation since DCT and inverse DCT operations do not require the knowledge of round keys. Similarly, the substitution can be guessed by $256^{M\times N}$ trials. Thus, the total guesses of round keys for one round is $(M\times N)! \times 256^{M\times N}$. If $M=N=4$, the approach to guess round keys will be with complexity greater than $2^{128}$. By increasing the number of rounds to 5 (for differential-like attack concern^40–42^), the proposed image encryption scheme shall provide sufficient security.

### Histogram analysis

**Figure 8:**
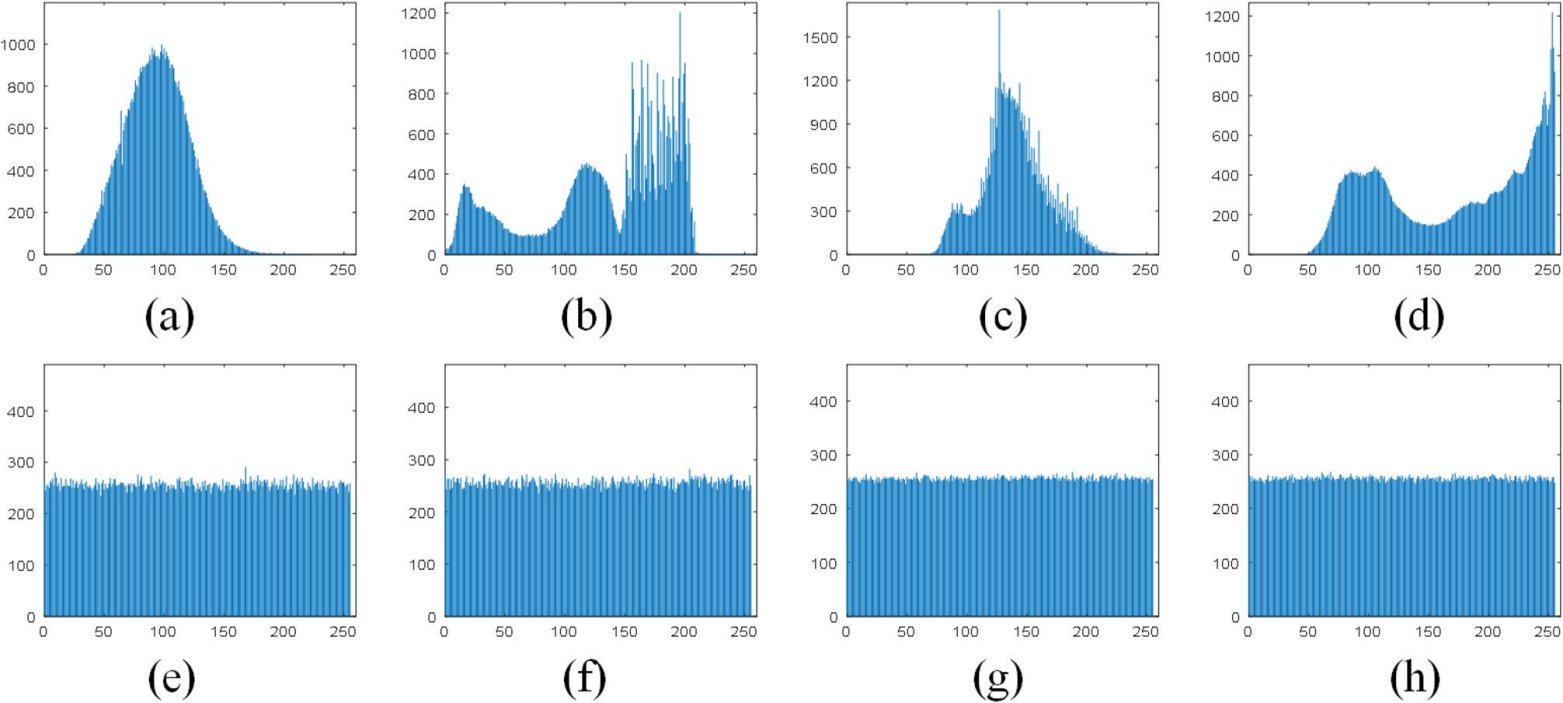
Histogram of plain images: (**a**) Grass; (**b**) Tree; (**c**) Pentagon; (**d**) Art; Histogram of encrypted images: (**e**) Grass; (**f**) Tree; (**g**) Pentagon; (**h**) Art.

The histogram is a basic attribute of a digital image, which reflects the statistical characteristics of the relationship between image gray level and image frequency. A good image encryption algorithm should make the pixels of the encrypted image more evenly distributed to effectively resist against statistical attacks. To verify the effectiveness of the proposed image encryption scheme, different images are tested. Figure 8a–d is the histogram corresponding to the original image, and Fig. 8e–h is the histogram corresponding to the encrypted image.

Besides, we also perform chi-square test to further evaluate the uniformity of histogram as shown in Eq. (9):

9 $$\begin{aligned} \chi ^2 = \sum _{L=0}^{255} \dfrac{(o_L-e_L)^2}{e_L}, \end{aligned}$$

where *L* is the intensity level, $o_L$ and $e_L$ are the observed reference and the expected reference of the gray level in the encrypted image, respectively. Table 3 shows the chi-square test results of the encrypted and plain images. Meanwhile, Table 4 presents the comparison of the chi-square value of the encrypted colour images between the proposed scheme with a recent proposed scheme^43^. The smaller the chi-square value, the more uniform the pixel distribution, thus the higher the safety.

**Table 3:** The chi-square test of images.

Image	Plain image	Encrypted image	Result
Grass	456678.6973	279.9434	Pass
Tree	225424.8183	275.7793	Pass
Pentagon	1993323.8129	246.2837	Pass
Art	710898.8340	233.0195	Pass
Sun	319180.2827	244.4310	Pass
Earth	382965.7695	256.9902	Pass

**Table 4:** The comparison of the chi-square test of color images.

Image	Airplane			Baboon		
	R	G	B	R	G	B
Plain image	15362	15059	25618	23763	35738	20450
This paper	261.96	232.68	270.45	262.13	218.36	244.97
^43^	265.19	245.62	287.91	287.56	227.89	259.78

### Correlation analysis

**Figure 9:**
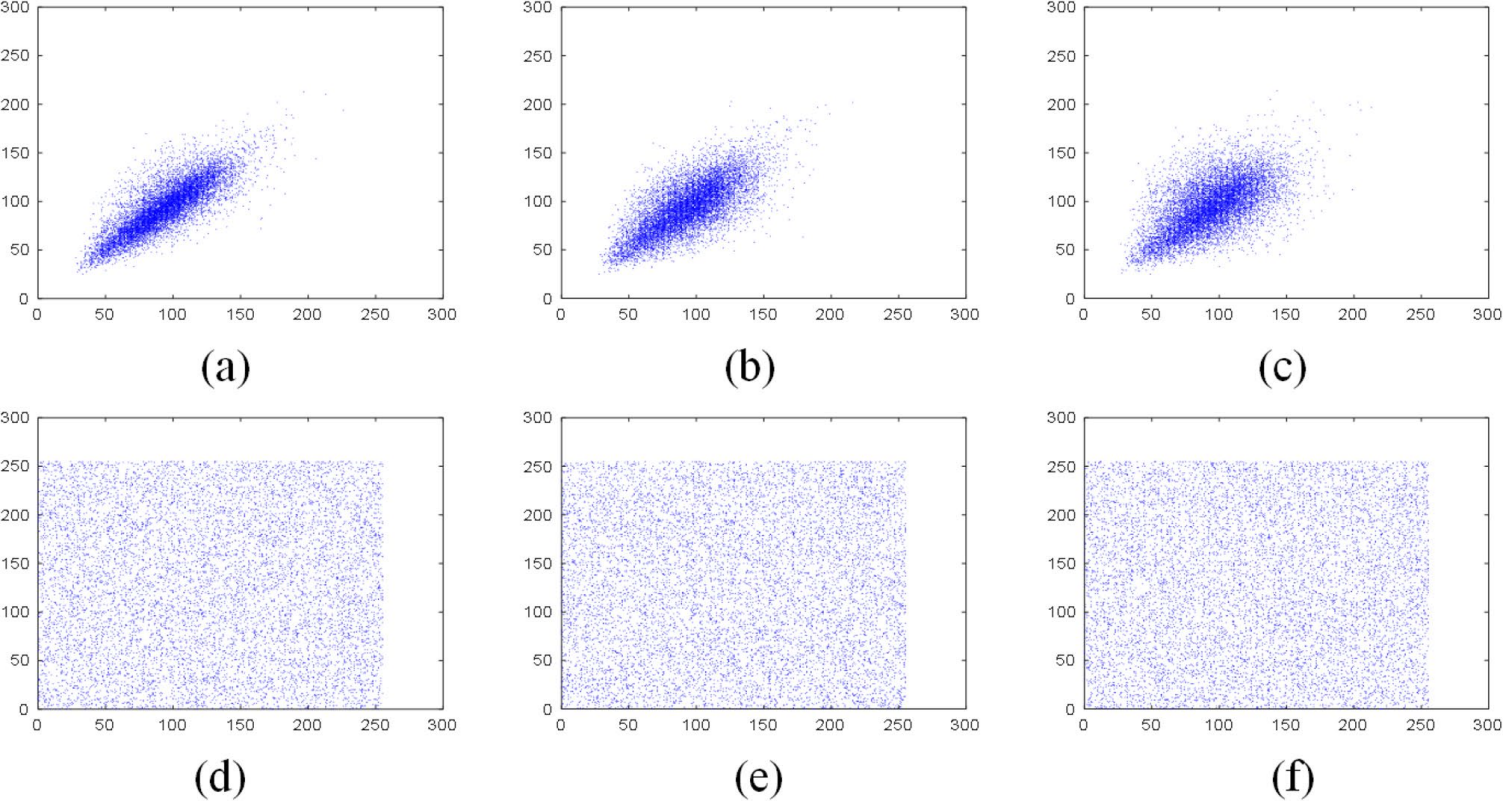
Correlation of adjacent pixels in the plain Grass image: (**a**) horizontal direction; (**b**) vertical direction; (**c**) diagonal direction; Correlation of adjacent pixels of the encrypted image: (**d**) horizontal direction; (**e**) vertical direction; (**f**) diagonal direction.

**Figure 10:**
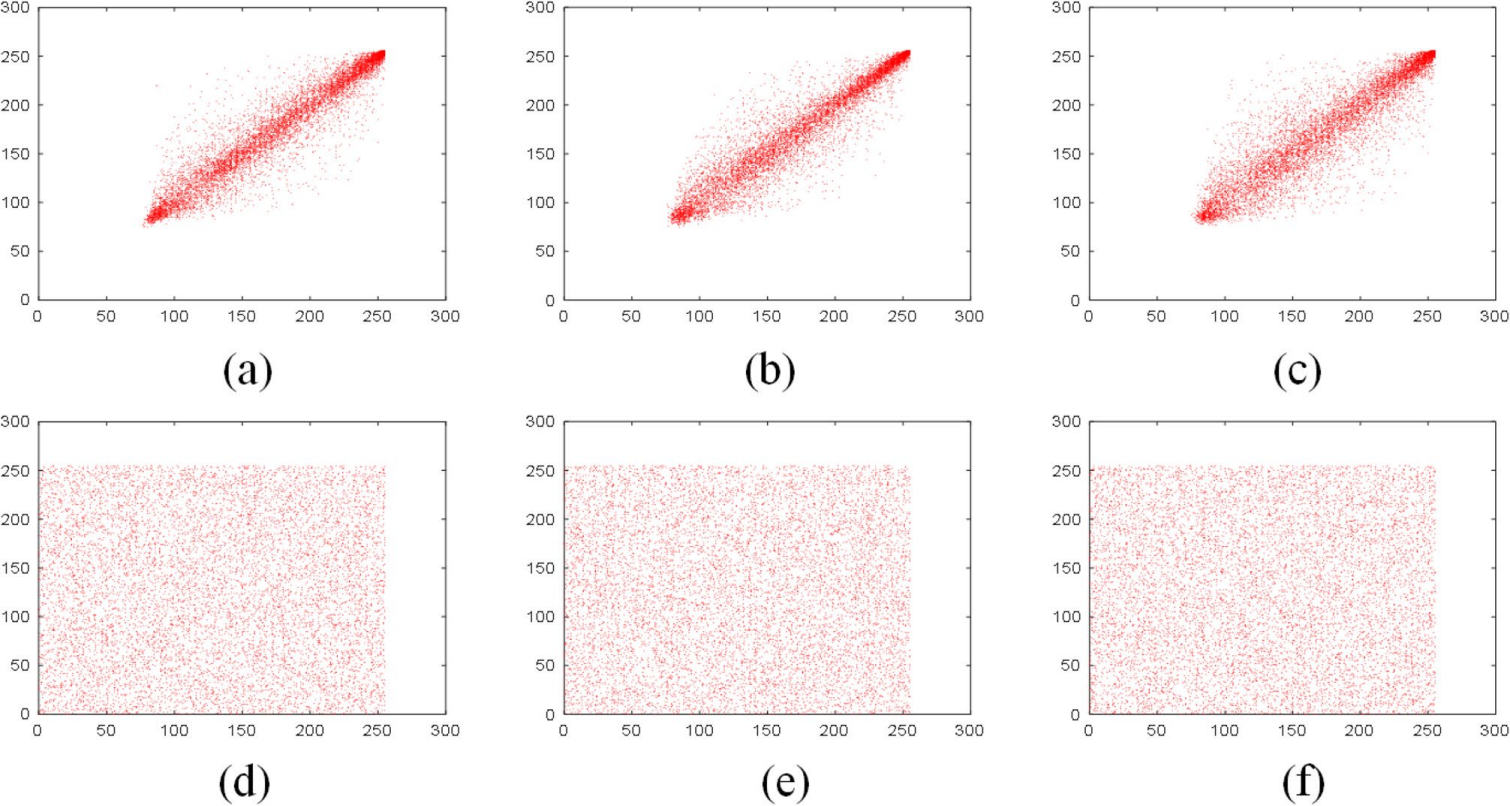
Correlation of adjacent pixels of R channel in plain Sun color image: (**a**) horizontal direction; (**b**) vertical direction; (**c**) diagonal direction; correlation of adjacent pixels of R channel of the encrypted image: (**d**) horizontal direction; (**e**) vertical direction; (**f**) diagonal direction.

**Figure 11:**
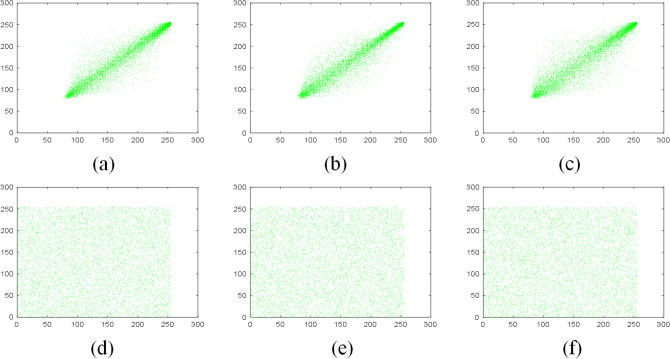
Correlation of adjacent pixels of G channel in plain Sun color image: (**a**) horizontal direction; (**b**) vertical direction; (**c**) diagonal direction; correlation of adjacent pixels of G channel of the encrypted image: (**d**) horizontal direction; (**e**) vertical direction; (**f**) diagonal direction.

**Figure 12:**
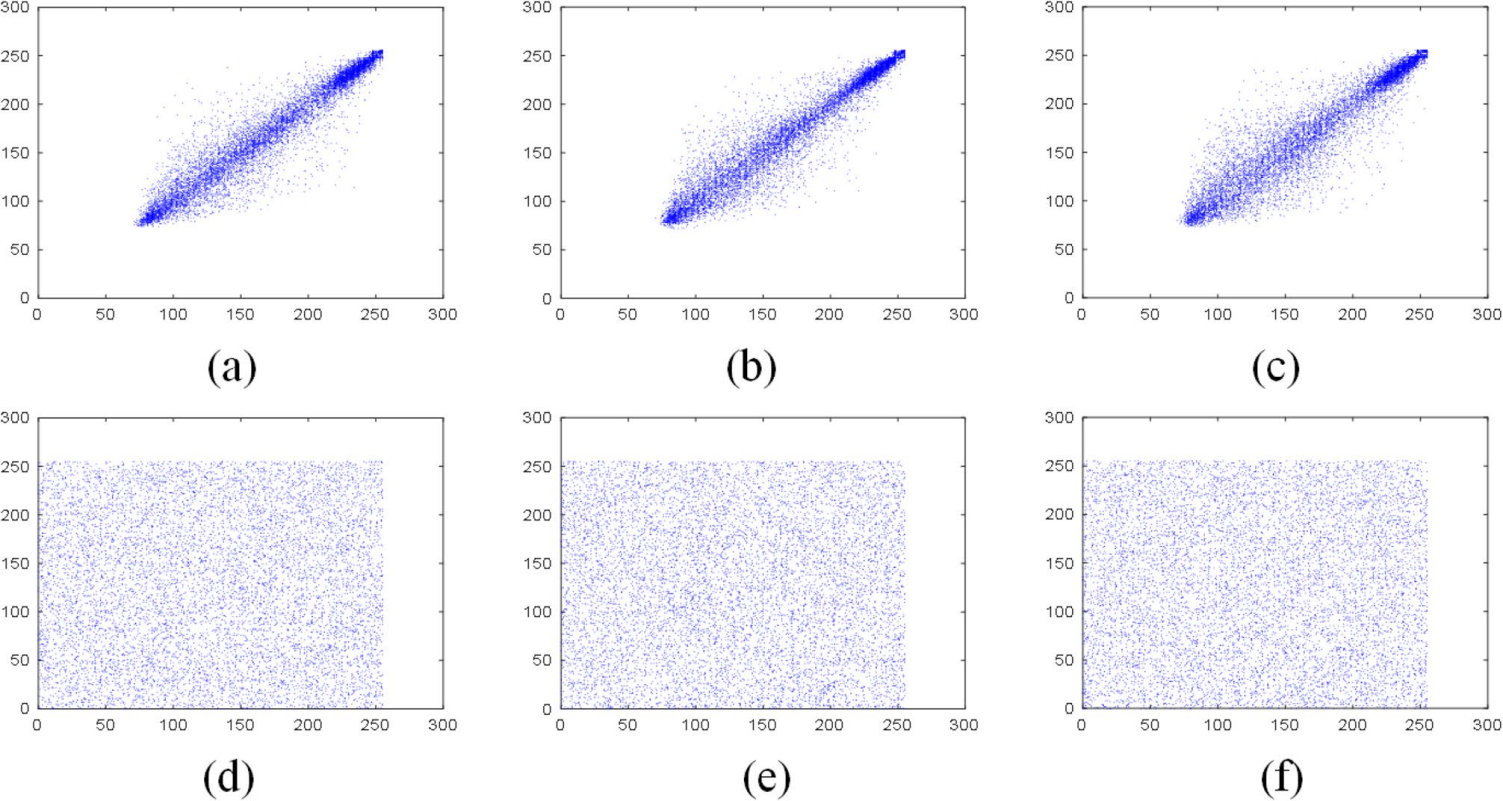
Correlation of adjacent pixels of B channel in plain Sun color image: (**a**) horizontal direction; (**b**) vertical direction; (**c**) diagonal direction; Correlation of adjacent pixels of B channel of the encrypted image: (**d**) horizontal direction; (**e**) vertical direction; (**f**) diagonal direction.

Since the original image has a strong correlation between adjacent pixels, the attacker may obtain meaningful information through the correlation of the pixels in the horizontal direction, vertical direction or diagonal direction. To measure the correlation coefficient, 10,000 adjacent pixel pairs of the plain image and the encrypted image are randomly selected from three different directions using Eq. (10).

10 $$\begin{aligned} R_{xy}&= \dfrac{cov(x,y)}{\sqrt{D(x)}\sqrt{D(y)}}, \end{aligned}$$

11 $$\begin{aligned} cov(x,y)&=\dfrac{1}{N}\sum _{i=1}^N(x_i-E(x))(y_i-E(y)), \end{aligned}$$

12 $$\begin{aligned} E(x)&= \dfrac{1}{N}\sum _{i=1}^Nx_i, \end{aligned}$$

13 $$\begin{aligned} D(x)&=\dfrac{1}{N}\sum _{i=1}^N(x_i-E(x))^2, \end{aligned}$$

where $R_{xy}$ represents the correlation coefficient, *x* and *y* are the gray values of two adjacent pixels, and *N* is the total pixels. Figure 9 shows the correlation of “Grass” grayscale image pixels and its encrypted image pixels in three different directions. Meanwhile, Figs. 10, 11 and 12 show the correlation of “Sun” color image pixels and its encrypted image pixels in three different directions and three different channels.

Tables 5 and 6 are the correlation coefficients of the grayscale and color images respectively. Table 7 compares the correlation coefficients of cipher images encrypted with different algorithms. It can be seen that the correlation coefficients of adjacent pixels in the plain images and encrypted images are close to 1 and 0 respectively, which indicates that the proposed scheme greatly reduces the correlation of adjacent pixels in the images. Besides, the pixels of the encrypted images are randomly distributed.

**Table 5:** The correlation coefficients of grayscale images.

Image	Plain image			Encrypted image		
	Horizontal	Vertical	Diagonal	Horizontal	Vertical	Diagonal
Grass	0.8129	0.7220	0.6439	0.0026	$-$ 0.0016	0.0047
Tree	0.9591	0.9667	0.9495	$-$ 0.0073	0.0094	0.0107
Pentagon	0.8567	0.8616	0.7960	0.0084	0.0026	0.0068
Art	0.9890	0.9914	0.9833	0.0112	0.0081	0.0035

**Table 6:** The correlation coefficients of color images.

Image	Direction	Plain image			Encrypted image		
		R	G	B	R	G	B
Sun	Horizontal	0.9575	0.9491	0.9581	0.0052	$-$ 0.0043	0.0093
	Vertical	0.9563	0.9511	0.9577	0.0075	0.0051	0.0114
	Diagonal	0.9340	0.9251	0.9397	$-$ 0.0026	$-$ 0.0007	0.0027
Earth	Horizontal	0.9723	0.9740	0.9594	0.0145	0.0035	0.0042
	Vertical	0.9676	0.9678	0.9539	0.0052	$-$ 0.0047	$-$ 0.0063
	Diagonal	0.9475	0.9502	0.9311	$-$ 0.0015	0.0071	0.0021

**Table 7:** The comparison of correlation coefficient of encrypted images.

Encrypted image	Reference	Correlation coefficients		
		Horizontal	Vertical	Diagonal
Baboon	This paper	0.0010	0.0023	0.0029
	^44^	0.0015	$-$ 0.0021	$-$ 0.0018
	^45^	0.0380	0.0097	$-$ 0.0081
	^46^	0.0016	0.0037	0.0053
	^47^	$-$ 0.0039	0.0120	0.0057
	^48^	0.0039	$-$ 0.0045	0.0039
Peppers	This Paper	$-$ 0.0005	0.0013	0.0017
	^44^	$-$ 0.0055	0.0025	0.0011
	^45^	$-$ 0.0175	$-$ 0.0021	0.0182
	^46^	$-$ 0.0013	0.0048	0.0016
	^49^	0.0018	0.0102	0.0016

### Information entropy

The information entropy reflects the average amount of information in an image, and the randomness of an image can be judged by the information entropy. For an image with 256 gray values, the ideal value of the global Shannon entropy is 8. The more uniform the gray value distribution, the stronger the randomness of the image. The information entropy can be computed using Eq. (14).

14 $$\begin{aligned} H(x) = -\sum _{i=0}^{N} p(x_i)log_2p(x_i), \end{aligned}$$

where $p(x_i)$ is the probability of the occurrence of $x_i$ and *N* is the total number of $x_i$. The global information entropy of the test image is shown in Table 8. It can be seen that the information entropy of encrypted image is very close to the ideal value, so the proposed image encryption scheme has high randomness.

**Table 8:** The global entropy of the images.

Image	Grass	Tree	Pentagon	Art	Sun	Earth
Plain image	6.73586	7.37607	6.73265	7.45320	7.29830	6.92869
Encrypted image	7.99923	7.99924	7.99983	7.99984	7.99933	7.99932

### Local entropy

The global entropy of image is a common analysis method in image encryption. However, the traditional Shannon entropy method has some shortcomings. For example, global entropy may fail to measure the true randomness of the image. The pixel information of the whole image needs to be measured through the global entropy with higher time complexity when the mage is with larger size^50^. As shown in Eq. (15)^51^, local information entropy is another evaluation method of image randomness, which reflects the randomness of the whole image through the randomly selected local random information.

15 $$\begin{aligned} \overline{H_{(k,TB)}} (S_i)= \sum _{i=1}^k \dfrac{H(S_i)}{k} \end{aligned}$$

for an image *S* and randomly select *k* non-overlapping blocks $S_i$. Besides, *TB* denotes the number of pixels in $S_i$ and $H(S_i)$ represents Shannon information entropy value. Table 9 shows the local information entropy value of the grayscale images and the color images (i.e. the mean value of the three different channels). It can be seen that the local information entropy of the image is above 7.95, which further reflects the randomness of the encrypted image.

**Table 9:** The local entropy of the images.

Image	Grass	Tree	Pentagon	Art	Sun	Earth
Encrypted image	7.95358	7.95390	7.95452	7.95496	7.95569	7.95457

### Differential attack analysis

According to the principles of cryptography, the encryption algorithm should be sufficiently sensitive to the changes of plaintext. The stronger the sensitivity, the stronger the ability to resist differential attacks. The number of pixels change rate (NPCR) and the unified average changing intensity (UACI) are important indicators to measure the resistance of image encryption algorithms to differential attacks. NPCR reflects the change rate of the gray value of the corresponding encrypted text, and UACI reflects the average change of the gray value. NPCR and UACI can be computed using Eqs. (16) and (17).

16 $$\begin{aligned} NPCR(C_1,C_2) = \sum _{i=1}^M\sum _{j=1}^N \dfrac{D(i,j)}{M\times N} \times 100\%, \end{aligned}$$

17 $$\begin{aligned} UACI(C_1,C_2) = \sum _{i=1}^M\sum _{j=1}^N \dfrac{|C_1(i,j)-C_2(i,j)|}{M\times N \times 255} \times 100\%, \end{aligned}$$

where $D(i,j) = 0$ if $C_1(i,j) = C_2(i,j)$; otherwise, 1. Besides, $C_1$ is the normal encrypted image, $C_2$ is the encrypted image when the value of a pixel in the original image changes, and $M\times N$ is the size of the image. Table 10 shows the NPCR and UACI values of grayscale encrypted images. Table 11 shows the comparison of NPCR and UACI values for different color images encrypted by different schemes. It can be seen from the table that both NPCR and UACI values pass the randomness test^52^, indicating that a modification of the pixel value will result in a completely different encrypted image. The results show that the proposed image encryption scheme can resist differential attack. Note that NPCR and UACR become two widely used metrics for the security analyses against differential attack in the image encryption community since these two metrics were introduced^53,54^.

**Table 10:** The average NPCR and UACI of grayscale images.

Image	NPCR (%)	UACI (%)
Grass	99.6052	33.4684
Tree	99.6132	33.4607
Pentagon	99.6213	33.4925
Art	99.6133	33.4558

**Table 11:** The comparison of NPCR and UACI values for color images.

Scheme	Image	NPCR (%)			UACI (%)		
		R	G	B	R	G	B
This paper	Peppers	99.6231	99.6286	99.6140	33.4674	33.5441	33.4935
	Baboon	99.6201	99.6445	99.6252	33.4552	33.4521	33.4367
	Average	99.6216	99.6366	99.6196	33.4613	33.4981	33.4651
^45^	Peppers	99.6315	99.6017	99.6380	33.5577	32.7183	33.5702
	Baboon	99.6246	99.5914	99.5972	32.6845	31.9555	33.4146
	Average	99.6281	99.5966	99.6176	33.1211	32.3369	33.4924
^55^	Peppers	99.6098	99.6218	99.5948	33.5012	33.4415	33.4536
	Baboon	99.6056	99.6164	99.6256	33.4478	33.4495	33.4401
	Average	99.6076	99.6191	99.6102	33.4745	33.4455	33.4469

### Key sensitivity analysis

**Figure 13:**
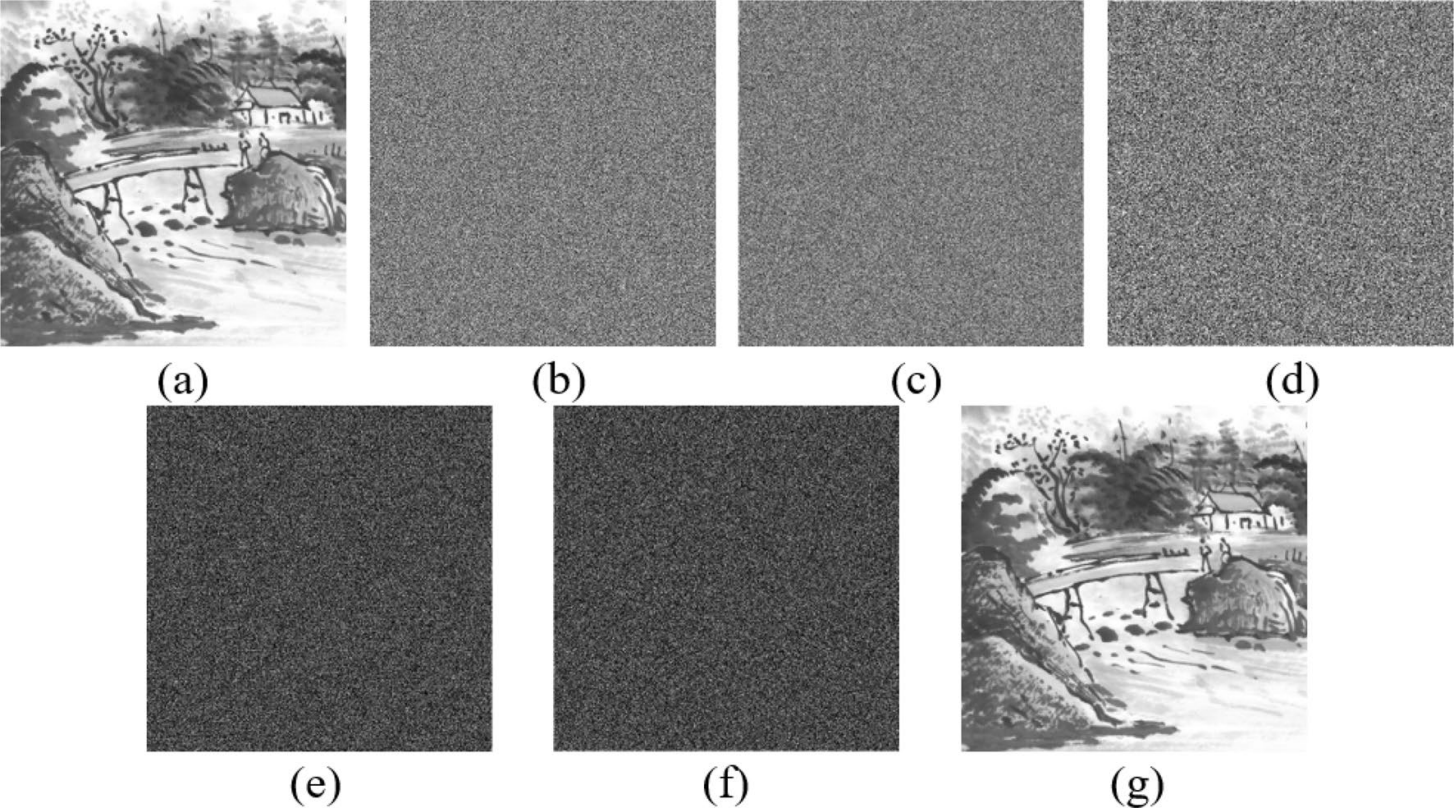
Key sensitivity test: (**a**) plain image; (**b**) encrypted image using $k_1$; (**c**) encrypted image using $k_2$; (**d**) difference image of (**b**,**c**); (**e**) decrypted image (**b**) using $k_2$; (**f**) decrypted image (**c**) using $k_1$; (**g**) decrypted image with correct key.

**Figure 14:**
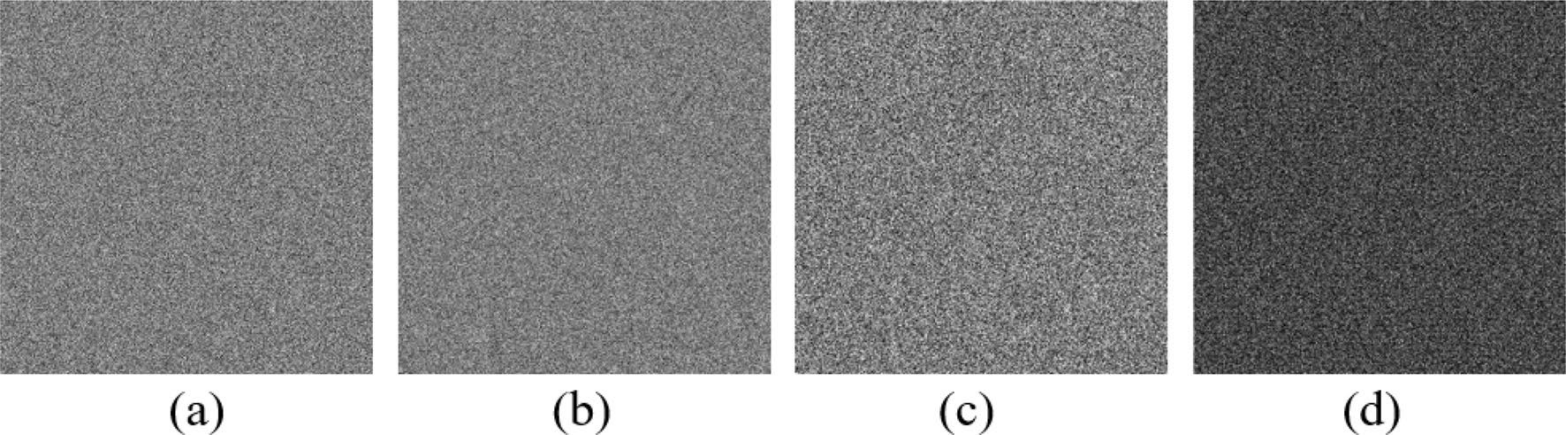
Key sensitivity test: (**a**) encrypted image with $\beta =6$; (**b**) encrypted image with $\beta =6.1$; (**c**) difference image of (**a**,**b**); (**d**) decrypted image (**a**) with $\beta =6.1$.

A good image encryption scheme should be highly sensitive to the changes of key. To measure the key sensitivity, assume two different initial value $x_0$ (denoted as $k_1$) and $x_0'$ (denoted as $k_2$) are selected to encrypt the image “Art”. Figure 13 shows the corresponding key sensitivity analysis results. It can be seen from Fig. 13d that there are great differences in the encrypted images with the changes of the initial value. As shown in Fig. 13e–g, only the correct key can recover the original image. Meanwhile, Fig. 14 shows the test results when the parameter of the quantum logistic map changes slightly. As a nutshell, the proposed image encryption scheme is sensitive to keys and can resist violent attacks.

### Cropping attack analysis

**Figure 15:**
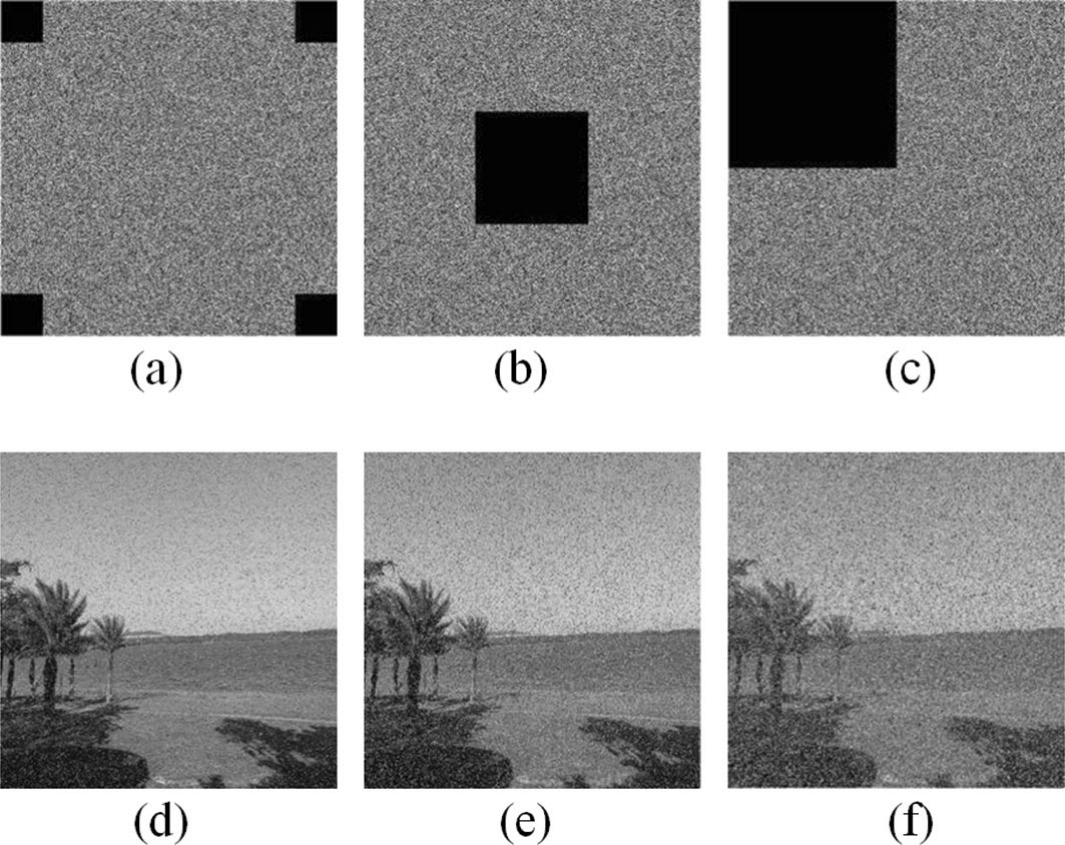
Tree image crop attack: (**a**) crop four small squares; (**b**) crop the middle part; (**c**) crop the upper left corner; (**d**) decryption of image (**a**); (**e**) decryption of image (**b**); (**f**) decryption of image (**c**).

**Figure 16:**
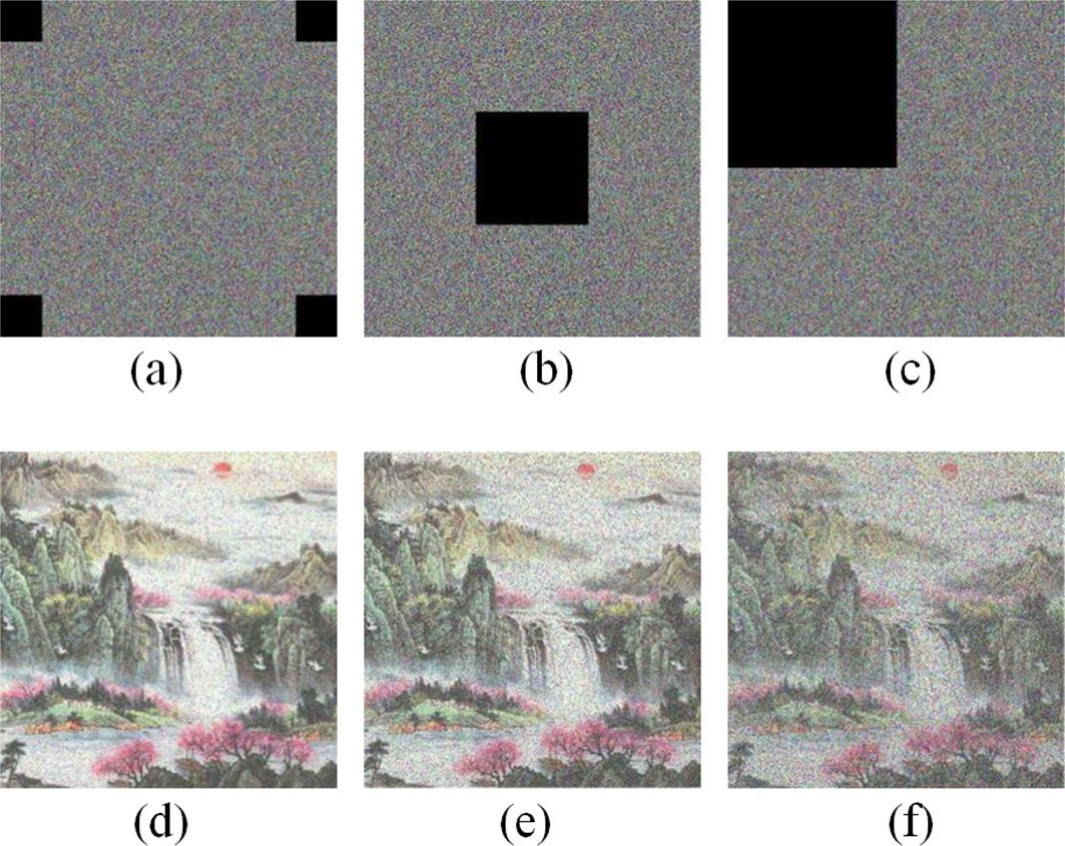
Color Sun image crop attack: (**a**) crop four small squares; (**b**) crop the middle part; (**c**) crop the upper left corner; (**d**) decryption of image (**a**); (**e**) decryption of image (**b**); (**f**) decryption of image (**c**).

When digital images are transmitted over the network, some data may be lost due to network congestion or malicious attack. When a part of the encrypted image is cropped, the pixel value of the corresponding part is replaced by zero. Figures 15 and 16 show the cropped decryption images with different data loss degrees. Although the decrypted image becomes more blur with the increase of crop area, the image is still visible. Therefore, the proposed image encryption scheme can resist shear attack to some extent.

### Noise attack

**Figure 17:**
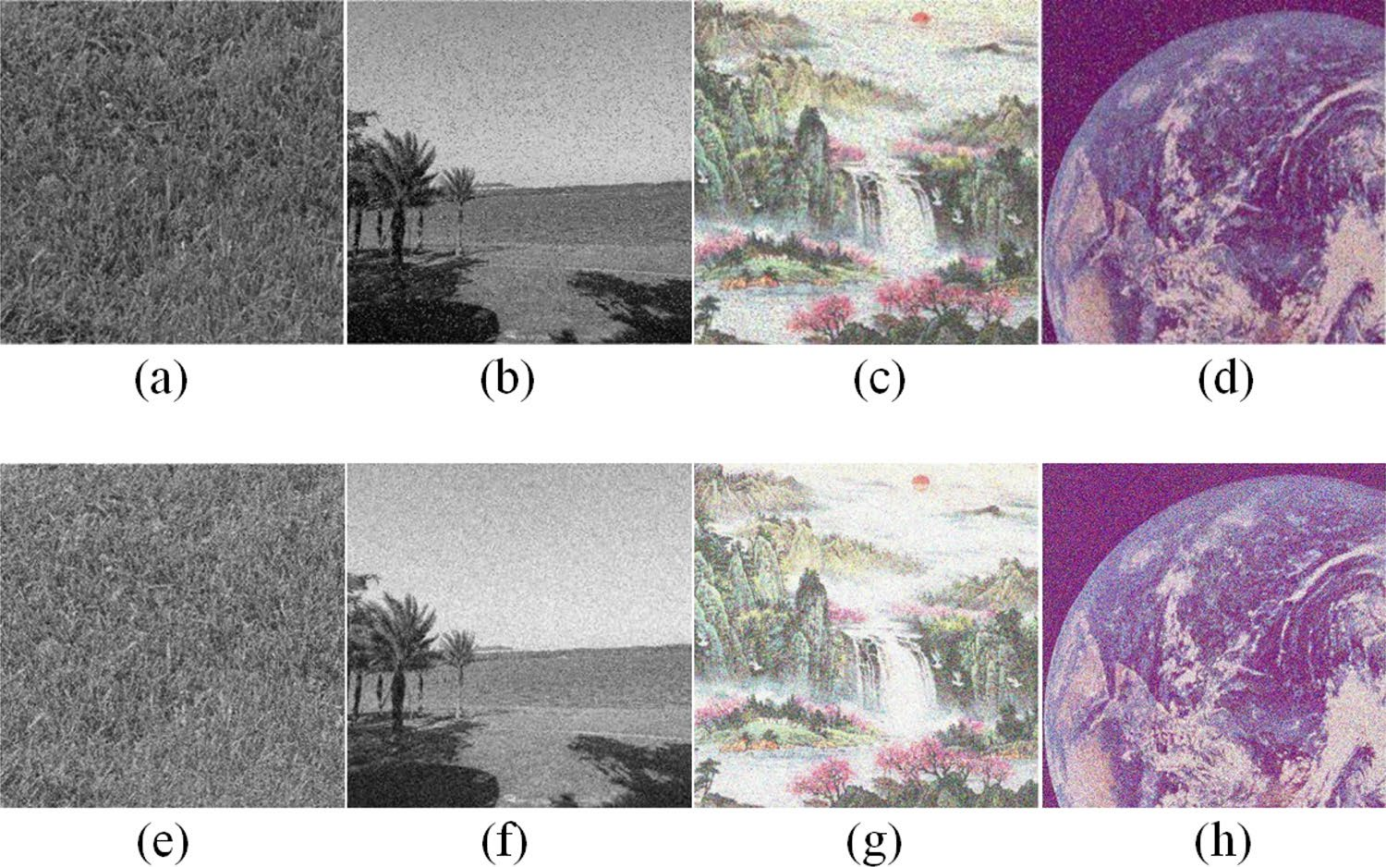
Decryption under salt and pepper noise: (**a**) Grass; (**b**) Tree; (**c**) Sun; (**d**) Earth; decryption of Gaussian noise: (**e**) Grass; (**f**) Tree; (**g**) Sun; (**h**) Earth.

Other than cropping attack, images are mainly polluted by noise during transmission. For example, pepper and salt noise (SPN), Gaussian noise (GN) and speckle noise (SN), which makes image recovery more difficult. Different noises with a mean value of 0 and variance of 0.006 are added to the encrypted image respectively, and the decrypted images are shown in Fig. 17. Although the quality of decrypted images decreases after affected by noise, the proposed image encryption scheme still resist noise attack to some extent.

### Comparison of information entropy

The encryption algorithm proposed in this paper is also applicable to color images. For the color image, it is divided into R, G, B channels where each channel is encrypted separately. Table 12 shows the information entropy of the proposed scheme as compared with other systems in three-channel color encrypted images. The results show that the gray value distribution of the proposed scheme is more uniform.

**Table 12:** The comparison between the proposed scheme and other image encryption schemes.

Image	Reference	Information Entropy		
		R	G	B
Baboon	This paper	7.9993	7.9993	7.9991
	^44^	7.9992	7.9993	7.9990
	^55^	7.9914	7.9915	7.9915
	^56^	7.9991	7.9991	7.9993
	^57^	7.9974	7.9992	7.9994
	^58^	7.9993	7.9993	7.9993
	^59^	7.9992	7.9993	7.9992
Peppers	This Paper	7.9993	7.9994	7.9992
	^23^	7.9986	7.9987	7.9985
	^49^	7.9979	7.9979	7.9979
	^55^	7.9911	7.9912	7.9915
	^56^	7.9989	7.9991	7.9989
	^57^	7.9972	7.9971	7.9969
	^58^	7.9992	7.9992	7.9993
	^59^	7.9992	7.9992	7.9993

## Conclusion

An image encryption scheme based on public key cryptosystems, quantum logistic map, discrete cosine transform and substitution–permutation network has been proposed. The public key cryptosystem is used to generate the initial values for the underlying quantum logistic map and avoid the need for a secure channel as compared to pure symmetric based image encryption. Meanwhile, DCT transforms the images into the frequency domain. SPN with five rounds provides adequate security against differential-like attacks and fast encryption which involves operations with lower computational complexity as compared to the asymmetric based image encryption. From the experimental results and various security analysis, it can be seen that the proposed image encryption is suitable for grayscale and color images, has a high sensitivity to plain text and keys, and secure against various attacks.
