# Author Correction: An image encryption scheme based on public key cryptosystem and quantum logistic map

**DOI:** 10.1038/s41598-021-87427-0

**Published:** 2021-04-14

**Authors:** Guodong Ye, Kaixin Jiao, Xiaoling Huang, Bok‑Min Goi, Wun‑She Yap

**Affiliations:** 1 Faculty of Mathematics and Computer Science, Guangdong Ocean University, Zhanjiang, 524088 China; 2 Lee Kong Chian Faculty of Engineering and Science, Universiti Tunku Abdul Rahman, Jalan Sungai Long, 43000 Cheras, Selangor Malaysia

Correction to: *Scientific Reports* 10.1038/s41598-020-78127-2, published online 03 December 2020

This Article contains errors in Reference 13, which is incorrectly given as:

Waseem, H. M. & Khan, M. Image encryption using quantum 3-d baker map and generalized gray code coupled with fractional chen’s chaotic system. Quantum Inf. Process. 19, 220. 10.1007/s11128-020-02724-3 (2020).

The correct reference is listed below:

Musanna, F. & Kumar, S. Image encryption using quantum 3-d baker map and generalized gray code coupled with fractional chen’s chaotic system. Quantum Inf. Process. 19, 220. 10.1007/s11128-020-02724-3 (2020).

